# Supplementary material for: Quantification of vascular networks in photoacoustic mesoscopy
Source: Photoacoustics. 2022 Apr 20;26:100357. doi: 10.1016/j.pacs.2022.100357 (PMC9095888; doi:10.1016/j.pacs.2022.100357)
Supplement: Supplementary Table 1 — Supplementary material [file mmc1.docx]

**Supplementary information for:**

Quantification of vascular networks in photoacoustic mesoscopy

**Authors:**

Emma L. Brown^1,2,#^, Thierry L. Lefebvre^1,2,#^, Paul W. Sweeney^1,2,#^, Bernadette J. Stolz^3^, Janek Gröhl^1,2^, Lina Hacker^1,2^, Ziqiang Huang^2 +^, Dominique-Laurent Couturier^2^, Heather A. Harrington^3^, Helen M. Byrne^3^ and Sarah E. Bohndiek^1,2^ *.

^1^ Department of Physics, University of Cambridge, JJ Thomson Avenue, Cambridge, CB3 0HE, U.K.

^2^ Cancer Research UK Cambridge Institute, University of Cambridge, Robinson Way, Cambridge, CB2 0RE, U.K.

^3^ Mathematical Institute, University of Oxford, Woodstock Road, Oxford, OX2 6GG, U.K.

+ Now at: European Molecular Biology Laboratory Imaging Centre, Heidelberg, Germany.

# These authors contributed equally

* Corresponding author: email [seb53@cam.ac.uk](mailto:seb53@cam.ac.uk), telephone +44 1223 337267.

**Keywords:** photoacoustic imaging, vasculature, segmentation, topology

**Supplementary Materials and Methods**

*1. String phantom preparation*

The string phantom used in this study was prepared by mixing 1.5 g agarose (Fluka Analytical, 05039-500G) in 97.3 mL deionised water in a glass media bottle and heated in a microwave until the solution turned clear. After cooling down the solution to 60°C, 2.08 mL of pre-warmed intralipid was added to generate a reduced scattering coefficient of 5.0 cm^-1^ according to a previously characterised recipe(Joseph et al., 2017). The mixture was poured into a 3D-printed phantom mould, which was designed in Autodesk Fusion 360 (San Rafael, CA, USA) and printed using an Anet A6 Printer with polylactic acid (PLA PRO 1.75mm Fluorescent Yellow PLA 3D Printer Filament, 832-0254, RS Components, UK) as a base material. **Supplementary Figure 2** shows the phantom mould with and without agar.

*2. 3D CNN for ROI delineation*

*2.1. Preparation of training data*

Image volumes consist of a series of 8-bit grayscale *Tiffs* (no compression) of 600 x 600 pixels in the XY-plane and a stack of 700 images in Z, with anisotropic voxels of size 20 x 20 x 4 μm^3^. Our dataset has a total of 166 PAI volumes, each paired with a corresponding binary semi-manually-annotated volume, where a voxel value of 0 and 255 indicates the background or tumour ROIs, respectively. The annotated volumes were generated by an experienced user, who first identified the top and bottom image containing the tumour in Z. Within these upper and lower bounds, ROIs were manually drawn in the XY plane on approximately 4 image slices. Bound by these data, a convex hull was extrapolated to approximate the ROI in the remaining image slices.

Prior to training, image volumes and binary masks were downsampled to an isotropic volume of 256 x 256 x 256 voxels to fit into computer memory. Data was locally standardised and normalised to a pixel range between 0 and 1 and the volumes randomly partitioned into training, validation, and testing subsets. Here, ~5% of images were allocated for testing, with the remaining portion split 80:20 for training and validation respectively (8 / 126 / 32 image volumes, respectively).

*2.2 Neural Network Architecture for ROI delineation*

The 3D CNN is based on the U-Net architecture(Ronneberger et al., 2015) extended for volumetric delineation(Çiçek et al., 2016). The structure consists of an encoder, which extracts spatial features from a 3D image volume, and a decoder, which constructs a segmentation map from these features (**Supplementary Figure 10**). The network architecture consists of five convolutional layers. The encoder path contains two 3 x 3 x 3 convolutions followed by a rectified linear unit (ReLU) activation for faster convergence and accuracy(Çiçek et al., 2016). Each ReLU activation is followed by 2 x 2 x 2 max pooling with strides of two in each dimension. For the 3^rd^, 4^th^ and 5^th^ layers, dropout is applied to reduce segmentation bias and ensure segmentation is performed utilising high-level features that may not have been considered in our semi-manual ROI annotations.

The decoder path consists of two 3 x 3 x 3 deconvolutions of strides of 2 in each dimension, followed by 3 x 3 x 3 convolutions, batch normalisation and ReLU activation. High-resolution features were provided via shortcut connections from the same layer in the encoder path. The final layer applied an additional 1 x 1 x 1 convolution followed by sigmoid activation to ensure the correct number of output channels and range of pixel values [0, 1]. The input layer is designed to take *n* grayscale (one channel) tumour volumes as input with a pre-defined volume (128 x 128 x 128 voxels in X, Y, Z-direction used here). The U-Net binary mask prediction contains an equal number of voxels as the input. The CNN was implemented in Keras(Chollet & Others, 2015) with the Tensorflow framework(Abadi et al., 2015). The model was trained and tested on a Dell Precision 7920 with a Dual Intel Xeon Gold 5120 CPU with 128 GB RAM and a NVIDIA Quadro GV100 32 GB GPU.

*2.3. Hyperparameter Optimisation*

Hyperparameters were optimised and evaluated using Talos(*Autonomio Talos*, 2019), a fully-automated hyperparameter tuner for Keras. A random search optimisation strategy was deployed using the quantum random method. Here, a probabilistic reduction scheme was used to reduce the number of parameter permutations by removing poorly performing hyperparameter configurations from the remaining search space after a predefined interval. The number of filters used ranged from 16 in the 1^st^ layer to 512 in the 5^th^. Dropout at a rate of 0.2 was applied in the 3^rd^, 4^th^ and 5^th^ layers. A Glorot uniform initialiser was used for all convolution and deconvolution layers. The model was trained using an Adam optimiser with learning and decay rates of 10^-5^ and 10^-8^, respectively, and the dice coefficient (F1)(Crum et al., 2006) used as the loss function.

*2.4. U-Net Training & Predictions*

Training was performed with a batch size of 3 image volumes for a total of 120 epochs (**Supplementary Figure 11A**). The fully-trained network achieved an accuracy of 88.3% and 87.3% on the training and validation sets respectively (**Supplementary Figure 11B**). Following training and test, we applied the CNN to the entire set of volumes to compare predictions of ROI volume to the ground truth (**Supplementary Figure 11C**). Blood volumes were then calculated within the predicted ROIs using the AT method and compared against the user annotations (**Supplementary Figure 11D**). We found a significant correlation between user annotated and predicted data for both ROI volume (Spearman’s rank correlation: r = 0.821, p < 0.0001) and blood volume (r = 0.958, p < 0.0001), indicating our CNN achieves sufficient performance against the experienced user to be applied for extracting tumours prior to testing the segmentation pipeline.

*3. Signal-to-noise ratio characterisation*

PAI quality pre-segmentation was quantified by measuring signal-to-noise ratio (SNR), defined as the mean of signal over the standard deviation of the background signal. For *in silico* and in phantom ground truth datasets, the mean of the signal was taken within the binary ground truth masks of the images and reported for different depths.

**Supplementary Tables**

**Supplementary Table 1:** Descriptions of our statistical and topological descriptors.

| **Descriptor** | **Description** |
| --- | --- |
| Connected Components, β_0_ | Number of 0-dimensional topological features, *i.e.* the number of subgraphs or clusters (vascular subnetworks). Values are normalised with respect to the total number of edges per segmented image volume. |
| Loops, β_1_ | Number of 1-dimensional topological features, *i.e.* the numbers of looping structures in vascular graph. Values are normalised with respect to the total number of edges per segmented image volume. |
| Sum-of-angles measure (SOAM) | The sum of angles between tangents to the curve taken at regular intervals normalised against vessel length, *i.e.* the average change in angle per unit length. |
| Chord-to-length ratio (CLR) | The ratio between the Euclidean distance connecting the two ends of a blood vessel and the length of the blood vessel, *e.g.* a straight vessel has a CLR equal to 1. |

**Supplementary Table 2:** Training and testing dataset split for random forest-based segmentation in ilastik.

| **Data** | **Ground truth labels** | **Training** | **Testing** |
| --- | --- | --- | --- |
| *In silico* | Original binary labels of L-net branches and surrounding background | 30 L-nets | 30 L-nets (data in **Figures 2** and **3**) |
| *In vitro* | Manual labelling of all XY slices containing strings and of surrounding background | 2 string phantom scans | 5 string phantom scans (data in **Figures 4** and **5**) |
| *In vivo* | Manual labelling of 10 XY slices per image at distributed depths and of surrounding background | 20 PDX tumour scans | 14 PDX tumour scans (data in **Figures 6-9**) |

**Supplementary Table 3.** Mean computation time in seconds for each segmentation method on *in silico, in vitro,* and *in vivo* data. Note: Segmentations were performed on a dual Intel Xeon E5-2623 v4 2.60 GHz quad-core processor and 64.0 GB of RAM.

| **Data** | **AT** | **AT+VF** | **RF** | **RF+VF** |
| --- | --- | --- | --- | --- |
| *In silico* | 7.4 | 198.1 | 191.1 | 381.8 |
| *In vitro* | 12.9 | 219.6 | 1280.0 | 1486.7 |
| *In vivo* | 38.4 | 1215.5 | 1500.7 | 2677.8 |

**Supplementary Table 4.** Absolute number of connected components for each L-Net skeleton generated from the ground truth and each segmentation method. Network names are organised based on number of recursive L-Net iterations and index, for example, ‘LNet_i4_0’ is the zeroth network of those with 4 iterations. Note, the number of known branching points is equal to number of iterations minus 3.

| **Name** | **Ground Truth** | **AT** | **AT+VF** | **RF** | **RF+VF** |
| --- | --- | --- | --- | --- | --- |
| LNet_i4_0 | 1 | 17 | 4 | 5 | 3 |
| LNet_i4_1 | 1 | 12 | 1 | 5 | 4 |
| LNet_i4_2 | 1 | 8 | 3 | 3 | 2 |
| LNet_i4_3 | 1 | 23 | 3 | 24 | 111 |
| LNet_i4_4 | 1 | 15 | 1 | 2 | 1 |
| LNet_i6_0 | 1 | 12 | 3 | 10 | 16 |
| LNet_i6_1 | 1 | 21 | 2 | 4 | 6 |
| LNet_i6_2 | 1 | 13 | 3 | 4 | 8 |
| LNet_i6_3 | 1 | 1 | 2 | 3 | 2 |
| LNet_i6_4 | 1 | 21 | 9 | 8 | 4 |
| LNet_i8_0 | 1 | 20 | 9 | 16 | 16 |
| LNet_i8_1 | 1 | 26 | 5 | 16 | 9 |
| LNet_i8_2 | 1 | 12 | 9 | 12 | 5 |
| LNet_i8_3 | 1 | 12 | 7 | 13 | 6 |
| LNet_i8_4 | 1 | 7 | 2 | 14 | 11 |
| LNet_i10_0 | 1 | 30 | 14 | 29 | 21 |
| LNet_i10_1 | 1 | 30 | 12 | 24 | 19 |
| LNet_i10_2 | 1 | 18 | 9 | 24 | 14 |
| LNet_i10_3 | 1 | 40 | 20 | 33 | 34 |
| LNet_i10_4 | 1 | 21 | 21 | 28 | 27 |
| LNet_i12_0 | 1 | 76 | 16 | 49 | 43 |
| LNet_i12_1 | 1 | 68 | 23 | 52 | 52 |
| LNet_i12_2 | 1 | 58 | 24 | 40 | 37 |
| LNet_i12_3 | 1 | 49 | 19 | 83 | 72 |
| LNet_i12_4 | 1 | 58 | 26 | 68 | 53 |
| LNet_i14_0 | 1 | 88 | 39 | 155 | 103 |
| LNet_i14_1 | 1 | 81 | 45 | 112 | 100 |
| LNet_i14_2 | 1 | 69 | 36 | 93 | 79 |
| LNet_i14_3 | 1 | 91 | 46 | 87 | 89 |
| LNet_i14_4 | 1 | 104 | 34 | 116 | 74 |

**Supplementary Table 5.** Absolute number of loops for each L-Net skeleton generated from the ground truth and each segmentation method. Network names are organised based on number of recursive L-Net iterations and index, for example, ‘LNet_i4_0’ is the zeroth network of those with 4 iterations. Note, the number of known branching points is equal to number of iterations minus 3.

| **Name** | **Ground Truth** | **AT** | **AT+VF** | **RF** | **RF+VF** |
| --- | --- | --- | --- | --- | --- |
| LNet_i4_0 | 0 | 46 | 0 | 2 | 2 |
| LNet_i4_1 | 0 | 27 | 0 | 4 | 16 |
| LNet_i4_2 | 0 | 42 | 0 | 0 | 8 |
| LNet_i4_3 | 1 | 45 | 13 | 67 | 86 |
| LNet_i4_4 | 0 | 41 | 0 | 1 | 11 |
| LNet_i6_0 | 0 | 54 | 1 | 12 | 27 |
| LNet_i6_1 | 1 | 28 | 0 | 13 | 11 |
| LNet_i6_2 | 1 | 63 | 7 | 22 | 72 |
| LNet_i6_3 | 0 | 6 | 0 | 0 | 0 |
| LNet_i6_4 | 2 | 47 | 0 | 9 | 8 |
| LNet_i8_0 | 4 | 68 | 1 | 33 | 22 |
| LNet_i8_1 | 2 | 21 | 0 | 4 | 1 |
| LNet_i8_2 | 2 | 53 | 0 | 2 | 11 |
| LNet_i8_3 | 1 | 86 | 0 | 8 | 13 |
| LNet_i8_4 | 1 | 40 | 0 | 1 | 0 |
| LNet_i10_0 | 4 | 0 | 0 | 0 | 0 |
| LNet_i10_1 | 20 | 14 | 0 | 0 | 1 |
| LNet_i10_2 | 9 | 20 | 0 | 0 | 0 |
| LNet_i10_3 | 9 | 33 | 0 | 14 | 12 |
| LNet_i10_4 | 11 | 7 | 0 | 1 | 1 |
| LNet_i12_0 | 73 | 9 | 4 | 24 | 12 |
| LNet_i12_1 | 123 | 37 | 5 | 25 | 25 |
| LNet_i12_2 | 106 | 32 | 7 | 35 | 36 |
| LNet_i12_3 | 30 | 4 | 1 | 1 | 1 |
| LNet_i12_4 | 62 | 2 | 1 | 8 | 13 |
| LNet_i14_0 | 353 | 16 | 9 | 18 | 13 |
| LNet_i14_1 | 426 | 43 | 15 | 58 | 43 |
| LNet_i14_2 | 395 | 19 | 9 | 19 | 20 |
| LNet_i14_3 | 376 | 74 | 15 | 58 | 51 |
| LNet_i14_4 | 304 | 29 | 12 | 20 | 29 |

**Supplementary Table 6.** The number of edges for each L-Net skeleton generated from the ground truth and each segmentation method. Network names are organised based on number of recursive L-Net iterations and index, for example, ‘LNet_i4_0’ is the zeroth network of those with 4 iterations. Note, the number of known branching points is equal to number of iterations minus 3.

| **Name** | **Ground Truth** | **AT** | **AT+VF** | **RF** | **RF+VF** |
| --- | --- | --- | --- | --- | --- |
| LNet_i4_0 | 3 | 160 | 4 | 19 | 10 |
| LNet_i4_1 | 3 | 122 | 3 | 24 | 59 |
| LNet_i4_2 | 3 | 144 | 3 | 5 | 32 |
| LNet_i4_3 | 7 | 177 | 49 | 247 | 420 |
| LNet_i4_4 | 3 | 136 | 3 | 9 | 38 |
| LNet_i6_0 | 15 | 217 | 18 | 63 | 122 |
| LNet_i6_1 | 18 | 134 | 12 | 55 | 60 |
| LNet_i6_2 | 19 | 222 | 34 | 94 | 236 |
| LNet_i6_3 | 15 | 37 | 14 | 15 | 14 |
| LNet_i6_4 | 21 | 167 | 11 | 50 | 43 |
| LNet_i8_0 | 65 | 267 | 34 | 172 | 130 |
| LNet_i8_1 | 65 | 139 | 19 | 77 | 57 |
| LNet_i8_2 | 69 | 227 | 35 | 68 | 85 |
| LNet_i8_3 | 62 | 315 | 33 | 85 | 92 |
| LNet_i8_4 | 62 | 148 | 18 | 44 | 37 |
| LNet_i10_0 | 238 | 114 | 46 | 113 | 101 |
| LNet_i10_1 | 251 | 161 | 54 | 116 | 111 |
| LNet_i10_2 | 196 | 164 | 43 | 100 | 86 |
| LNet_i10_3 | 179 | 241 | 64 | 197 | 183 |
| LNet_i10_4 | 253 | 138 | 69 | 151 | 138 |
| LNet_i12_0 | 687 | 211 | 97 | 332 | 282 |
| LNet_i12_1 | 669 | 283 | 103 | 296 | 296 |
| LNet_i12_2 | 698 | 310 | 130 | 388 | 349 |
| LNet_i12_3 | 794 | 188 | 84 | 182 | 197 |
| LNet_i12_4 | 662 | 150 | 79 | 249 | 246 |
| LNet_i14_0 | 2359 | 317 | 175 | 524 | 396 |
| LNet_i14_1 | 2226 | 463 | 262 | 642 | 591 |
| LNet_i14_2 | 2037 | 261 | 158 | 461 | 427 |
| LNet_i14_3 | 1707 | 459 | 174 | 506 | 461 |
| LNet_i14_4 | 1936 | 345 | 153 | 462 | 460 |

**Supplementary Table 7.** The number of nodes for each L-Net skeleton generated from the ground truth and each segmentation method. Network names are organised based on number of recursive L-Net iterations and index, for example, ‘LNet_i4_0’ is the zeroth network of those with 4 iterations. Note, the number of known branching points is equal to number of iterations minus 3.

| **Name** | **Ground Truth** | **AT** | **AT+VF** | **RF** | **RF+VF** |
| --- | --- | --- | --- | --- | --- |
| LNet_i4_0 | 4 | 131 | 8 | 22 | 11 |
| LNet_i4_1 | 4 | 107 | 4 | 25 | 47 |
| LNet_i4_2 | 4 | 110 | 6 | 8 | 26 |
| LNet_i4_3 | 7 | 155 | 39 | 204 | 445 |
| LNet_i4_4 | 4 | 110 | 4 | 10 | 28 |
| LNet_i6_0 | 16 | 175 | 20 | 61 | 111 |
| LNet_i6_1 | 18 | 127 | 14 | 46 | 55 |
| LNet_i6_2 | 19 | 172 | 30 | 76 | 172 |
| LNet_i6_3 | 16 | 32 | 16 | 18 | 16 |
| LNet_i6_4 | 20 | 141 | 20 | 49 | 39 |
| LNet_i8_0 | 62 | 219 | 42 | 155 | 124 |
| LNet_i8_1 | 64 | 144 | 24 | 89 | 65 |
| LNet_i8_2 | 68 | 186 | 44 | 78 | 79 |
| LNet_i8_3 | 62 | 241 | 40 | 90 | 85 |
| LNet_i8_4 | 62 | 115 | 20 | 57 | 48 |
| LNet_i10_0 | 235 | 144 | 60 | 142 | 122 |
| LNet_i10_1 | 232 | 177 | 66 | 140 | 129 |
| LNet_i10_2 | 188 | 162 | 52 | 124 | 100 |
| LNet_i10_3 | 171 | 248 | 84 | 216 | 205 |
| LNet_i10_4 | 243 | 152 | 90 | 178 | 164 |
| LNet_i12_0 | 615 | 278 | 109 | 357 | 313 |
| LNet_i12_1 | 547 | 314 | 121 | 323 | 323 |
| LNet_i12_2 | 593 | 336 | 147 | 393 | 350 |
| LNet_i12_3 | 765 | 233 | 102 | 264 | 268 |
| LNet_i12_4 | 601 | 206 | 104 | 309 | 286 |
| LNet_i14_0 | 2007 | 389 | 205 | 661 | 486 |
| LNet_i14_1 | 1801 | 501 | 292 | 696 | 648 |
| LNet_i14_2 | 1643 | 311 | 185 | 535 | 486 |
| LNet_i14_3 | 1332 | 476 | 205 | 535 | 499 |
| LNet_i14_4 | 1633 | 420 | 175 | 558 | 505 |

**Supplementary Figures**


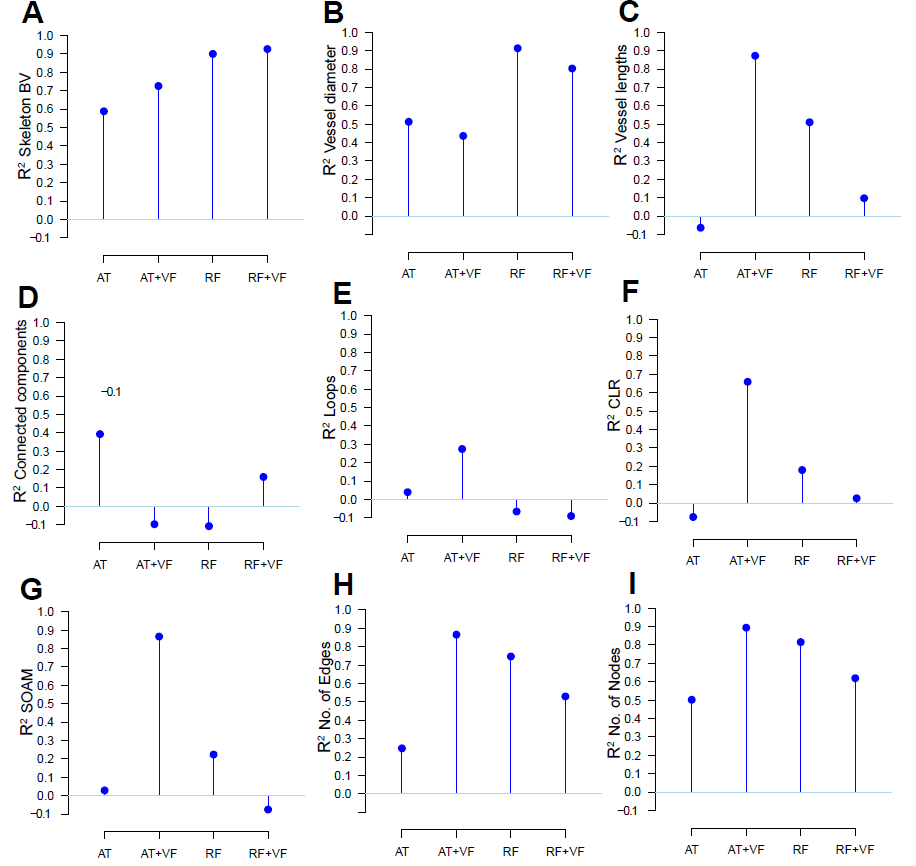


**Supplementary Figure 1. Random forest classifier segments PAI networks with high accuracy while autothresholding with vesselness filtering preserves network structure.** Bar plot for R^2^ values calculated to compare the strength of relationship between the segmented networks (AT, AT+VF, RF or RF+VF) and ground-truth L-nets for the following metrics: (A) Normalised skeleton blood volume (BV), (B) Vessel diameters, µm, (C) Vessel lengths, µm, (D) Connected components, (E) Loops, (F) chord-to-length ratio (CLR), (G) sum-of-angle measure (SOAM), (H) Number of Edges and (I) Number of Nodes.

**Supplementary Figure 2. Photographs of the string phantom.** (A) 3D-printed mould (7.4 x 7.4 cm, wall thickness: 4 mm) with the embedded strings and (B) with the agar gel. The top string was positioned at 0.5 mm from the agar surface, the middle one at 1 mm, and the bottom one at 2 mm depth.


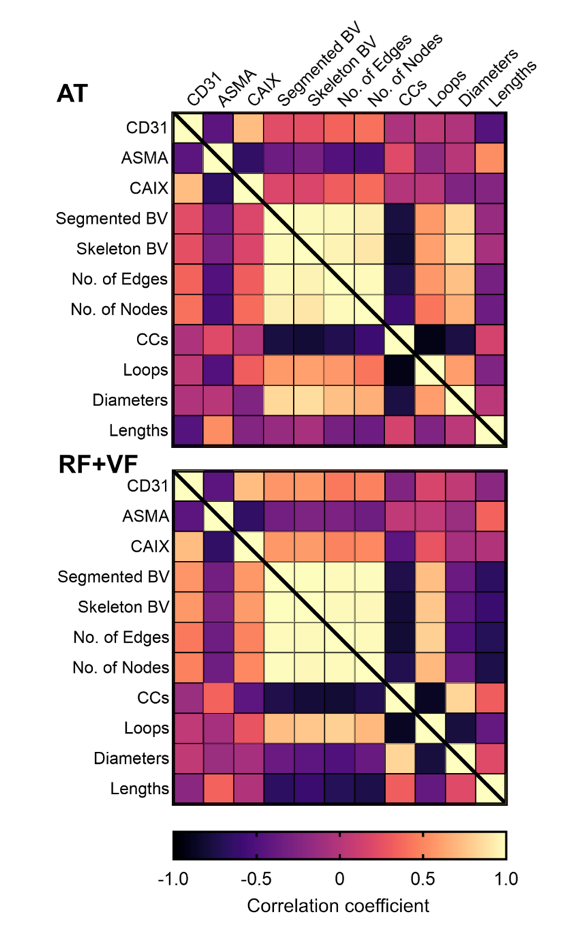


**Supplementary Figure 3. Correlation between blood volume and statistical and topological *in vivo* metrics with *ex vivo* IHC in AT and RF+VF segmented networks.** Matrix of correlation coefficients for AT (top) and RF+VF (bottom) segmented networks. Pearson or spearman coefficients are used as appropriate, depending on data distribution. Note that none of the coefficients are significant for AT networks (p>0.05). For RF+VF, CD31 staining area and CAIX significantly correlated with segmented (p=0.04 and p=0.03 respectively) and skeletonised blood volume (p=0.03 for both).


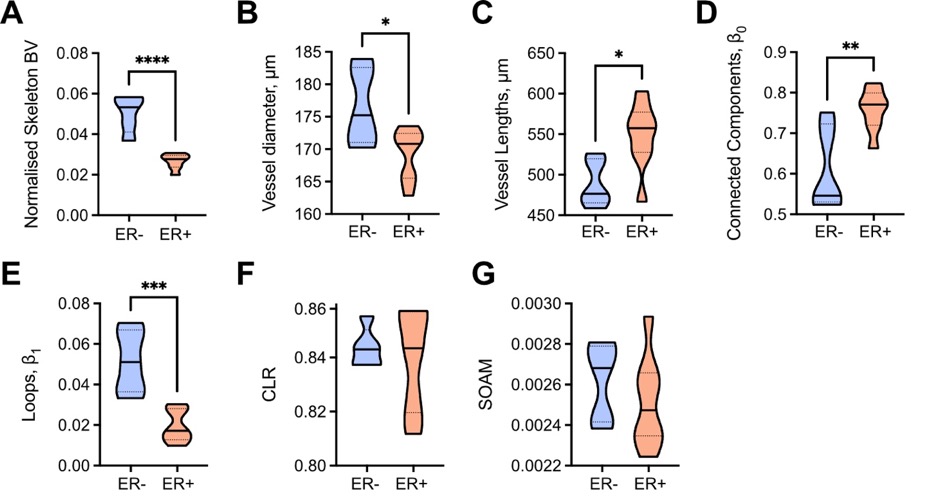


**Supplementary Figure 4. Statistical and topological analyses of AT+VF segmentation masks comparing ER- and ER+ tumours.** (A-G) Abbreviations defined: blood volume (BV), chord-to-length ratio (CLR), sum-of-angle measure (SOAM). Data are represented by truncated violin plots with interquartile range (dotted black) and median (solid black). Comparisons between ER- and ER+ tumours made with unpaired t-test. *= p<0.05, **=p<0.01, ***=p<0.001, ****=p<0.0001.


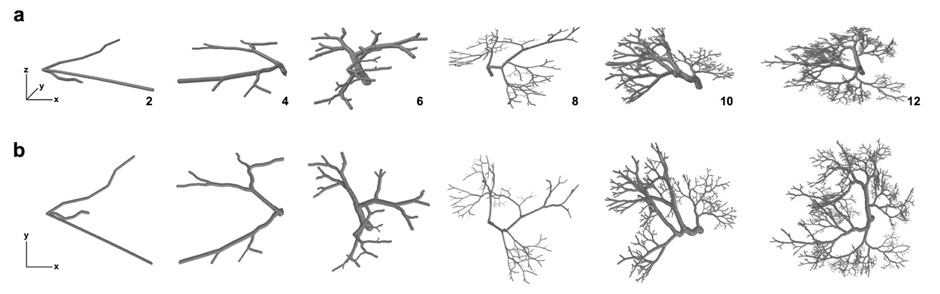


**Supplementary Figure 5. Generation of Lindenmayer System (L-System) vascular networks.** (A) Segmented views of L-System vasculatures for an increasing number of branching generations (left to right; number of generations indicated). (B) Projected view in the (X,Y) plane of the architectures shown in (A).

**Supplementary Figure 6. RSOM data pre-processing in MATLAB.** Mean Intensity Projection 2D view of an example RSOM tumour dataset along Z axis (A-C) and Y axis (D-F) axis. From left to right: raw data (A,D), high-pass filtered data (B,E), Wiener filtered data (C,F). The images are processed sequentially through this pipeline, using high-pass filtering to remove echo noises and low-pass adaptive Wiener filtering to further remove stochastic noise in the datasets. (G) Image after MATLAB pre-processing. (H) Image after background correction with rolling ball subtraction in Fiji. The periodical horizontal line artefacts are mostly removed after background correction. All images are 6 x 6 mm.


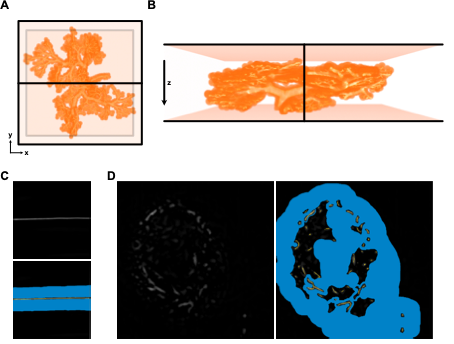


**Supplementary Figure 7. Labelling of photoacoustic data for random forest classifier training with ilastik.** (A,B) Labels for the full vascular architecture of a given L-net were used for training of ilastik. The region of the L-net within 10 voxels of the vessels was labelled as background (dark orange) in addition to a three voxel thick planes (shown in black). The first was located parallel to the z-axis, with the remaining two perpendicular at the top and bottom of the image volume. (C) Labelling of string volumes and (D) of PDX tumour vessels for ilastik training. For (C) and (D) background was labelled as blue and vessels labelled as yellow on 2D slices throughout the 3D volume stack. All images are 6 x 6 mm.

**Supplementary Figure 8. Median filtering of segmented RSOM images.** A 3D rendering of the exemplar RSOM dataset (6 x 6 x 2.5 mm in X, Y and Z dimensions) used in Supplementary Figure 6 is shown. (A) Autothresholded dataset. (B) Autothresholded dataset after 3D Median filtering, to remove impulse noise.

**Supplementary Figure 9. 3D U-Net architecture.** The blue boxes indicate feature maps with the number of channels denoted above. The input and output image volumes consist of 128 x 128 x 128 voxels. Concat = concatenation, Conv = convolution, ReLu = rectified linear unit, Deconv = deconvolution.


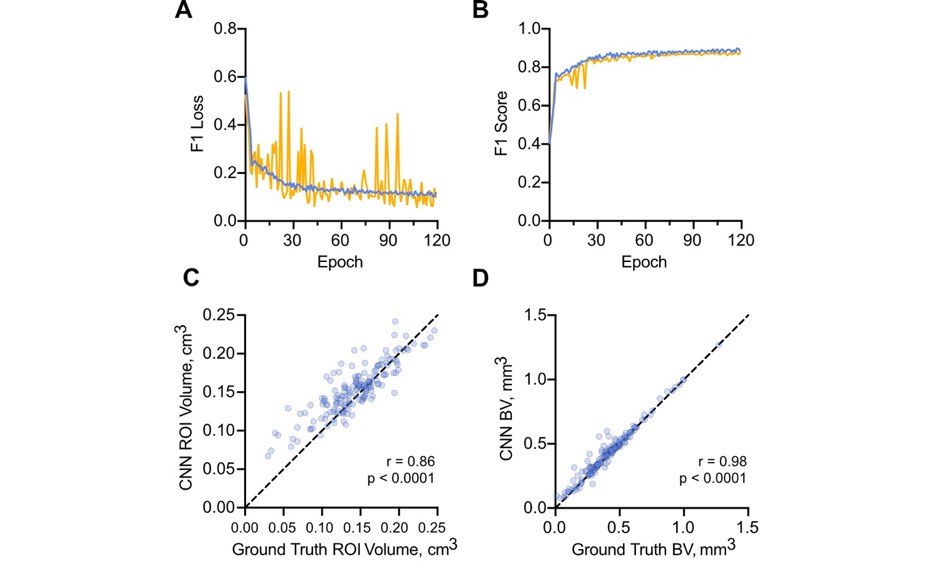


**Supplementary Figure 10. U-Net training metrics and predictions from the fully-trained architecture.** Training metrics: (A) F1 loss and (B) F1 score for the training (blue) and validation (orange) datasets. (C) Region-of-interest volumes calculated from the ground truth (GT) versus the U-Net mask. (D) Computed blood volumes using the ground truth and U-Net ROI estimations from (C). Note, the lines in (C) and (D) indicate a 1-to-1 relationship, and blood volumes in (B) were calculated using our auto-thresholding segmentation method.
